# Interactome Data and Databases: Different Types of Protein Interaction

**DOI:** 10.1002/cfg.377

**Published:** 2004-03

**Authors:** Javier De Las Rivas, Alberto de Luis

**Affiliations:** Cancer Research Center (CIC, USAL-CSIC) University of Salamanca and CSIC Campus Miguel de Unamuno Salamanca E37007 Spain

## Abstract

In recent years, the biomolecular sciences have been driven forward by overwhelming advances in new biotechnological high-throughput experimental methods and bioinformatic genome-wide computational methods. Such breakthroughs are producing huge amounts of new data that need to be carefully analysed to obtain correct and useful scientific knowledge. One of the fields where this advance has become more intense is the study of the network of ‘protein–protein interactions’, i.e. the ‘interactome’. In this short review we comment on the main data and databases produced in this field in last 5 years. We also present a rationalized scheme of biological definitions that will be useful for a better understanding and interpretation of ‘what a protein–protein interaction is’ and ‘which types of protein–protein interactions are found in a living cell’. Finally, we comment on some assignments of interactome data to defined types of protein interaction and we present a new bioinformatic tool called APIN (Agile Protein Interaction Network browser), which is in development and will be applied to browsing protein interaction databases.


**Comparative and Functional Genomics**
*Comp Funct Genom* 2004; **5**: 173–178.
Published online in Wiley InterScience (www.interscience.wiley.com). **DOI:** 10.1002/cfg.377

**Conference Review**

### Interactome data and databases: different types of protein interaction

Javier De Las Rivas* and Alberto de Luis
*Cancer Research Center (CIC, USAL-CSIC), University of Salamanca and CSIC, Campus Miguel de Unamuno, E37007 Salamanca, Spain*

**Correspondence to: Javier De Las Rivas, Cancer Research Center (CIC, USAL-CSIC), University of Salamanca and CSIC, Campus Miguel de Unamuno, E37007 Salamanca, Spain. E-mail: jrivas@usal.es*

Received: 12 November 2003
Revised: 10 December 2003
Accepted: 18 December 2003

### Abstract

**In recent years, the biomolecular sciences have been driven forward by overwhelming advances in new biotechnological high-throughput experimental methods and bioinformatic genome-wide computational methods. Such breakthroughs are producing huge amounts of new data that need to be carefully analysed to obtain correct and useful scientific knowledge. One of the fields where this advance has become more intense is the study of the network of 'protein–protein interactions', i.e. the 'interactome'. In this short review we comment on the main data and databases produced in this field in last 5 years. We also present a rationalized scheme of biological definitions that will be useful for a better understanding and interpretation of 'what a protein–protein interaction is' and 'which types of protein–protein interactions are found in a living cell'. Finally, we comment on some assignments of interactome data to defined types of protein interaction and we present a new bioinformatic tool called APIN (Agile Protein Interaction Network browser), which is in development and will be applied to browsing protein interaction databases. Copyright © 2004 John Wiley & Sons, Ltd.**

**Keywords:** **bioinformatics; databases; interactome; protein interaction; protein network**

### Experimental approaches to protein–protein interaction networks

In recent years, work in genomics, proteomics and bioinformatics is producing vast amounts of data that have to be stored and well organized in biological databases. In this respect, one of the most productive areas has been that focused on protein–protein interactions in whole cells [8]. Several scientific breakthroughs have been achieved in this field, with the first global experimental analyses of protein–protein interactions made in yeast (*Saccharomyces cerevisiae*) using the *two-hybrid system* [17,29]. This system provides a large-scale experimental approach to determine whether or not each pair of proteins from the yeast proteome physically interact. Another large-scale experimental approach based on proteomics technology, also applied to yeast, is the systematic *isolation of multiprotein complexes* previously tagged, followed by *tandem-affinity purification* and *mass spectrometry* to identify the proteins present in each isolated complex [12]. A different experimental procedure that has been developed to explore large-scale protein binary interactions is *protein microarrays*. A first high-density yeast proteome microarray composed of 5800 fusion proteins was built and used to identify novel calmodulin-binding and phospholipid-binding proteins [37]. Finally, other high-throughput technologies used to determine the gene expression profile of thousands of genes are *DNA microarrays* [26,36] and *serial analysis of gene expression* (SAGE) [34]. These techniques measure mRNA levels, producing expression profiles that indicate those proteins that are co-expressed and that are probably working together in a cellular state or in a specific cellular response. DNA microarrays usually provide better genome-wide information; however, one attractive feature of SAGE when compared to microarrays is its

Copyright © 2004 John Wiley & Sons, Ltd.

ability to quantify gene expression without prior sequence information [34].

### Computational approaches to protein–protein interaction networks

In parallel to the experimental approaches, a series of computational bioinformatic methods have been designed to predict protein–protein interactions. These methods are based on the analysis of different genome-wide characteristics. The first methods that were published look for features at the genome level: conservation of *gene order and neighbourhood* [5,28] and identification of *domain fusion events* for orthologous genes [9,10,20]. Other methods explore features at the proteome level: comparison of the *phylogenetic profiles* of orthologous proteins in complete proteomes (i.e. patterns of presence/absence of orthologous proteins) [6, 11, 24]; identification of proteins with similar *phylogenetic trees*, using multiple sequence alignments of families of homologous proteins [13, 23, 25]; and identification of *correlated mutations* between the multiple sequence alignments of pairs of proteins [21, 22].

All of the computational methods described are based on the hypothesis that it is possible to predict the interaction of two proteins when such proteins form an 'associated pair' which interacts at a certain biomolecular and cellular level. These associated pairs of proteins undergo a common evolutionary pressure and such co-evolution can be tracked at different structural and functional levels. In this way, these bioinformatic methods give additional information about the functional properties of the proteins and they are powerful tools to draw protein–protein interaction networks [30]. The computational methods can be applied to predicted protein interactions across whole genomes/proteomes and in this way they allow multiple genome comparisons.

### Databases of Protein Interactions

As a consequence of experimental and bioinformatic approaches providing data about interacting proteins on a genome- and proteome-wide scale, several research groups have also made an important effort in designing and setting up databases that include computer-controlled information about these 'interactomes'.

The most significant public databases of protein interactions are: Biomolecular Interaction Network Database (BIND [1]); Database of Interacting Proteins (DIP [33]); the General Repository for Interaction Datasets (GRID [4]); Molecular Interactions Database (MINT [35]); and a database of predicted functional associations among genes/proteins (STRING [31]). There are some other biological websites that include information from several of the databases listed above, or tools to view and browse such data. A review about the state of molecular biology databases is published each year in the journal *Nucleic Acids Research*. In the 2003 issue [3] the state of the BIND, GRID and STRING databases was reported, indicating full activity and good development of the data that they contain. DIP and MINT were reported on in the 2002 issue [2]. The structure and type of data that these databases contain is similar, but not the same. Most include the yeast interactome, which is becoming a reference set, together with interactome data from other species and some curated data coming from small-scale experiments (see review [32]). DIP is probably the most highly curated database of protein interactions. Curation in DIP is done manually by expert curators and also automatically using computational approaches [7]. DIP includes catalogues of experimentally determined interactions between proteins and it combines information from a variety of sources to create a consistent dataset of protein–protein interactions [33].

### What do we mean by protein interaction?

Intuitively, the definition of protein interaction in its more restrictive meaning would only involve the interaction produced by physical contact between the surfaces of two proteins. But most of the methods currently used have a bias towards the detection of higher levels of relation or association between proteins. Such protein relations can be very different: inclusion in *multiprotein complexes*, common *cellular compartments*, same *signalling pathway*, same *metabolic pathway, co-expression, genetic co-regulation*, or even molecular *co-evolution*. These

Copyright © 2004 John Wiley & Sons, Ltd. 

multiple types of protein relations result in a muddling datascape. In this way, the complete protein network (the interactome) that operates in a cell is complicated not only by the large number of proteins involved, but also by the range of distinct types of protein interactions, and if we want to infer some biological meaning they can not be mixed together in general datasets. An English saying points out that 'generalize' is very close to 'generally lies', and this can happen if protein interaction data are stored in a database without discrimination or discernment about the type of interaction. The 'generalize' type of thinking is also present in the current scientific trend for speaking about 'gen*ome*', 'prote*ome*', 'interact*ome*', 'metabol*ome*' and so on. This way of defining biological entities is neat when the object of definition has a clear biological meaning, i.e. it refers to a clear biological function, as is the case for 'genome'. But, in the case of 'interactome' such a definition is more complex, since it includes several 'omes' that have very different biological meanings and functions. The complexity of the interactome has not yet been clearly addressed in scientific forums, and just recently some authors have started to analyse the multidimensional structure of networks of protein–protein interactions [18, 27].

### Different types of protein interaction

The existence of different types of interactions has been shown when comparative analyses of global approaches to protein–protein interactions have been made [32]. Legrain and co-workers have discussed the limitations of methods for massive determination of protein interactions, showing that there is a low degree of overlap between the interactions determined by different methods [19]. With this in mind, other authors have designed computational tools to try to assess the reliability of data from high-throughput screening approaches [7]. Both questions, i.e. the low overlapping of interactome datasets and the low reliability of high-throughput screening methods, are correlated with the problem underlined above: protein–protein interaction data obtained with different methods explore different types of interaction/association.

Under these arguments, it seems evident that there is a need to better define what types of protein–protein associations can be found in a living cell. We undertake that definition, trying to cover the main biological features that involve protein–protein association in a unicellular living organism. In this way we can distinguish three levels of association, with several sublevels:

1. *Co-interacting proteins*, defined as *physical interaction*:
   (a) *Permanent* interaction: proteins forming a stable protein complex that carries out a biomolecular role (structural or functional). These proteins are *protein subunits* of the complex and they work together. Examples include ATPase subunits, subunits of the nuclear pore, and ribosomal proteins within the S and L elements of the ribosome.
   (b) *Transient* interaction: proteins that come together in certain cellular states to undertake a biomolecular function. Examples include the DNA replicative complex, and most of the proteins involved in signal transduction cascades.
2. *Correlated proteins*, defined as proteins that are involved in the *same biomolecular activity* but that do not interact physically:
   (a) *Metabolic* correlation: proteins involved in the same metabolic pathway. These proteins are mostly *enzymes*. Examples include Krebs cycle enzymes, and prostaglandin synthesis enzymes.
   (b) *Genetic* correlation: proteins that are encoded by co-expressed or co-regulated genes. These could be called *operon-type* proteins. Examples include enzymes that regulate the glycolytic pathway, and proteins that regulate a phase of the cell cycle.
3. *Co-located proteins*, defined as proteins that work in the same *cellular compartment*:
   (a) *Soluble* location: proteins placed in the same cellular soluble space. Examples include proteins in the lysosome, proteins in the mitochondrial stroma, and proteins in the endoplasmic reticulum.
   (b) *Membrane* location: proteins placed in the same cellular membrane. Examples include receptors in the plasma membrane, transporters in the mitochondrial membrane, and membrane translocation complexes.

Copyright © 2004 John Wiley & Sons, Ltd. 

It is clear that two proteins can be linked by more than one of the defined types of association. Moreover, one particular protein can be related to several others by different types of association. Therefore, the defined types are not exclusive. This fact reflects the complexity of protein networks, but these definitions provide a foundation for better understanding of what we mean by interactome.

### Some assignments of interactome data to defined types of protein interaction

The given definitions should improve the analysis of data about interactomes. Some assignments can be done based on the properties of the experimental technique used to produce the data. So, the *two-hybrid system* and *protein microarrays* produce mainly data about transient co-interacting proteins (class 1b). The two-hybrid system is limited only to pairwise interactions. The *purification of complexes and mass spectrometry* technique reveals mostly stable co-interacting proteins (class 1a). *Expression microarrays* measure mRNA levels and reveal co-expressed proteins that may also be co-regulated (class 2b).

Assignments for the bioinformatic methods have been made by quantifying *a posteriori* the type of protein interaction that each method produces (see reviews [16,30]). In this way, quantitative evaluation by Huynen and collaborators [15] found that 66–80% of the associations detected by the *conservation of gene order* method correspond to physical interactions, and about 13% to metabolic correlation. Of the associations detected as *gene fusion events*, about 66% correspond to physical interactions and about 15% to metabolic correlation. The associations detected by *phylogenetic profiles* correspond to physical interactions in about 33% of cases and to metabolic correlation in 33% of cases.

The number of collections of interacting proteins (i.e. known interactomes) is still small and

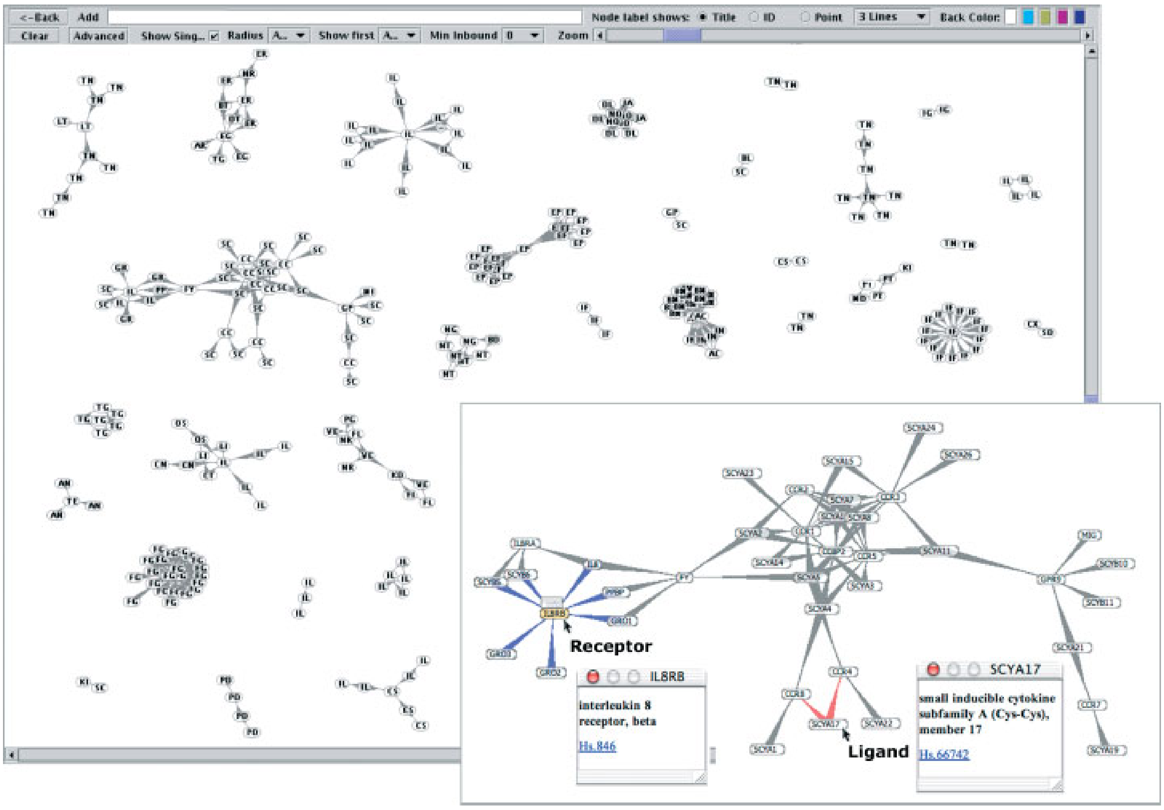

**Figure 1.** A view of the APIN tool displaying a subset from DIP database corresponding to all known human ligand–receptor interactions [14]. The colour and direction of the links indicate whether a protein is a ligand or a receptor. The number of links shows the number of proteins that bind a certain receptor or ligand

Copyright © 2004 John Wiley & Sons, Ltd. 

therefore these assignments are preliminary and partial. However, it seems clear that databases of protein interactions should incorporate not only new collections of interacting proteins but also good definitions and annotation about the types of protein interaction that they include in each case. Due to the fact that such annotations are impossible in many cases of high-throughput methods, new efforts to develop automated computational tools to compare datasets, and to assess their accuracy and coverage, are needed.

### APIN, an agile protein interaction network browser

As a first step to facilitate the understanding of protein-protein interaction networks we have developed a bioinformatic tool called APIN (Agile Protein Interaction Network browser). This tool allows users to view and browse the nodes and links/edges of a protein interaction database (Figure 1). The nodes are the proteins and the links or edges are the types of interaction. APIN is based on a JAVA applet (TouchGraph) and displays the data stored in a MySQL database of interactions. The APIN browser reads data from the database and displays them in an interactive customizable way, like a browser. In this way, using an intuitive and clear interface one can navigate into complex interactome databases focusing on some areas, or on some specific proteins, by applying restrictions to the queried data. The tool is still in early development and we are implementing its capacity and power to browse over complex protein interaction networks, including the three levels of association described.

### Acknowledgement

We thank the support and research funding provided by "Castilla y Leòn" Local Government (JCyL project ref. SA104/03) and the Spanish Ministry of Health (MSyC project ref. P1030920).

### References

1. Bader GD, Betel D, Hogue CW. 2003. BIND: the Biomolecular Interaction Network Database. *Nucleic Acids Res* **31**: 248–250.
2. Baxevanis AD. 2002. The Molecular Biology Database Collection: 2002 update. *Nucleic Acids Res* **30**: 1–12.
3. Baxevanis AD. 2003. The Molecular Biology Database Collection: 2003 update. *Nucleic Acids Res* **31**: 1–12.
4. Breitkreutz BJ, Stark C, Tyers M. 2003. The GRID: the General Repository for Interaction Datasets. *Genome Biol* **4**: R23.
5. Dandekar T, Snel B, Huynen M, Bork P. 1998. Conservation of gene order: a fingerprint of proteins that physically interact. *Trends Biochem Sci* **23**: 324–328.
6. De Las Rivas J, Lozano JJ, Ortiz AR. 2002. Comparative analysis of chloroplast genomes: functional annotation, genome-based phylogeny, and deduced evolutionary patterns. *Genome Res* **12**: 567–583.
7. Deane CM, Salwinski L, Xenarios I, Eisenberg D. 2002. Protein interactions: two methods for assessment of the reliability of high throughput observations. *Mol Cell Proteomics* **1**: 349–356.
8. Drewes G, Bouwmeester T. 2003. Global approaches to protein–protein interactions. *Curr Opin Cell Biol* **15**: 199–205.
9. Enright AJ, Iliopoulos I, Kyrpides NC, Ouzounis CA. 1999. Protein interaction maps for complete genomes based on gene fusion events. *Nature* **402**: 86–90.
10. Enright AJ, Ouzounis CA. 2001. Functional associations of proteins in entire genomes by means of exhaustive detection of gene fusions. *Genome Biol* **2**: 0034.
11. Gaasterland T, Ragan MA. 1998. Microbial genescapes: phyletic and functional patterns of ORF distribution among prokaryotes. *Microb Comp Genom* **3**: 199–217.
12. Gavin AC, Bosche M, Krause R, *et al.* 2002. Functional organization of the yeast proteome by systematic analysis of protein complexes. *Nature* **415**: 141–147.
13. Goh CS, Bogan AA, Joachimiak M, Walther D, Cohen FE 2000. Co-evolution of proteins with their interaction partners. *J Mol Biol* **299**: 283–293.
14. Graeber TG, Eisenberg D. 2001. Bioinformatic identification of potential autocrine signaling loops in cancers from gene expression profiles. *Nature Genet* **29**: 295–300.
15. Huynen M, Snel B, Lathe W III, Bork P. 2000. Predicting protein function by genomic context: quantitative evaluation and qualitative inferences. *Genome Res* **10**: 1204–1210.
16. Huynen MA, Snel B, von Mering C, Bork P. 2003. Function prediction and protein networks. *Curr Opin Cell Biol* **15**: 191–198.
17. Ito T, Chiba T, Ozawa R, *et al.* 2001. A comprehensive two-hybrid analysis to explore the yeast protein interactome. *Proc Natl Acad Sci USA* **98**: 4569–4574.
18. Jeong H, Mason SP, Barabasi AL, Oltvai ZN. 2001. Lethality and centrality in protein networks. *Nature* **411**: 41–42.
19. Legrain P, Wojcik J, Gauthier JM. 2001. Protein–protein interaction maps: a lead towards cellular functions. *Trends Genet* **17**: 346–352.
20. Marcotte EM, Pellegrini M, Ng HL, *et al.* 1999. Detecting protein function and protein–protein interactions from genome sequences. *Science* **285**: 751–753.
21. Pazos F, Helmer-Citterich M, Ausiello G, Valencia A. 1997. Correlated mutations contain information about protein–protein interaction. *J Mol Biol* **271**: 511–523.
22. Pazos F, Valencia A. 2002. *In silico* two-hybrid system for the selection of physically interacting protein pairs. *Proteins* **47**: 219–272.

Copyright © 2004 John Wiley & Sons, Ltd.

23. Pazos F, Valencia A. 2001. Similarity of phylogenetic trees as indicator of protein–protein interaction. *Protein Eng* **14**: 609–614.
24. Pellegrini M, Marcotte EM, Thompson MJ, Eisenberg D, Yeates TO. 1999. Assigning protein functions by comparative genome analysis: protein phylogenetic profiles. *Proc Natl Acad Sci USA* **96**: 4285–4288.
25. Ramani AK, Marcotte EM. 2003. Exploiting the co-evolution of interacting proteins to discover interaction specificity. *J Mol Biol* **327**: 273–284.
26. Slonim DK. 2002. From patterns to pathways: gene expression data analysis comes of age. *Nature Genet* **32**(suppl): 502–508.
27. Spirin V, Mirny LA. 2003. Protein complexes and functional modules in molecular networks. *Proc Natl Acad Sci USA* **100**: 12 123–12 128.
28. Tamames J, Casari G, Ouzounis C, Valencia A. 1997. Conserved clusters of functionally related genes in two bacterial genomes. *J Mol Evol* **44**: 66–73.
29. Uetz P, Giot L, Cagney G, *et al*. 2000. A comprehensive analysis of protein–protein interactions in *Saccharomyces cerevisiae*. *Nature* **403**: 623–627.
30. Valencia A, Pazos F. 2002. Computational methods for the prediction of protein interactions. *Curr Opin Struct Biol* **12**: 368–373.
31. von Mering C, Huynen M, Jaeggi D, *et al*. 2003. STRING: a database of predicted functional associations between proteins. *Nucleic Acids Res* **31**: 258–261.
32. Xenarios I, Eisenberg D. 2001. Protein interaction databases. *Curr Opin Biotechnol* **12**: 334–339.
33. Xenarios I, Salwinski L, Duan XJ, *et al*. 2002. DIP, the Database of Interacting Proteins: a research tool for studying cellular networks of protein interactions. *Nucleic Acids Res* **30**: 303–305.
34. Ye SQ, Usher DC, Zhang LQ. 2002. Gene expression profiling of human diseases by serial analysis of gene expression. *J Biomed Sci* **9**: 384–394.
35. Zanzoni A, Montecchi-Palazzi L, Quondam M, *et al*. 2002. MINT: a Molecular INTeraction database. *FEBS Lett* **513**: 135–140.
36. Zhang MQ. 1999. Large-scale gene expression data analysis: a new challenge to computational biologists. *Genome Res* **9**: 681–688.
37. Zhu H, Bilgin M, Bangham R, *et al*. 2001. Global analysis of protein activities using proteome chips. *Science* **293**: 2101–2105.

Copyright © 2004 John Wiley & Sons, Ltd. 
